# Epigenetic regulation of CD271, a potential cancer stem cell marker associated with chemoresistance and metastatic capacity

**DOI:** 10.3892/or.2014.3569

**Published:** 2014-10-24

**Authors:** SULAN LI, DONGLI YUE, XINFENG CHEN, LIPING WANG, JIEYAO LI, YU PING, QUN GAO, DAN WANG, TENGFEI ZHANG, FENG LI, LI YANG, LAN HUANG, YI ZHANG

**Affiliations:** 1 Biotherapy Center, The First Affiliated Hospital of Zhengzhou University, Zhengzhou, Henan 450052, P.R. China; 2 Department of Radiology, The First Affiliated Hospital of Zhengzhou University, Zhengzhou, Henan 450052, P.R. China; 3 Department of Oncology, The First Affiliated Hospital of Zhengzhou University, Zhengzhou, Henan 450052, P.R. China; 4 School of Life Sciences, Zhengzhou University, Zhengzhou, Henan 450052, P.R. China; 5 Institute of Clinical-Medicine, The First Affiliated Hospital of Zhengzhou University, Zhengzhou, Henan 450052, P.R. China

**Keywords:** esophageal squamous cell carcinoma, cancer stem cell, CD271, DNA methylation

## Abstract

Cancer stem cells (CSCs) are considered to be the cause of tumor initiation, metastasis and recurrence. Additionally, CSCs are responsible for the failure of chemotherapy and radiotherapy. The isolation and identification of CSCs is crucial for facilitating the monitoring, therapy or prevention of cancer. We aimed to identify esophageal squamous cell carcinoma (ESCC) stem-like cells, the epigenetic mechanism and identify novel biomarkers for targeting ESCC CSCs. Sixty-three paired ESCC tissues and adjacent non-cancerous tissues were included in this study. CD271, which was identified as the CSC marker for melanoma, was assessed using quantitative PCR (qPCR). Using flow cytometry, we isolated CD271^+^ cells comprising 7.5% of cancer cells from the KYSE70 cell line. Sphere formation and anchorage-independent growth were analyzed in CD271^+^ and CD271^−^ cancer cells, respectively. qPCR was used to detect stem-related genes and CCK-8 was performed to analyze the sensitivity to chemotherapy in the two groups. Bisulfite genomic sequencing was used to analyze the methylation status. CD271 expression was significantly higher in ESCC tissues than in adjacent non-cancerous tissues. Compared with CD271^−^ cancer cells, CD271^+^ cancer cells showed a higher ability of sphere and colony formation, a high level expression of stem-related gene, and resistance to chemotherapy. The expression of CD271 was induced by a demethylation agent. In conclusion, CD271^+^ ESCC cells possess stem-like properties. CD271 can potentially act as a prognostic marker for ESCC, whose expression is regulated epigenetically.

## Introduction

Esophageal squamous cell carcinoma (ESCC) is a lethal malignancy with a 5-year survival rate of 26.2% due to late diagnosis, rapid growth and metastasis (1). Thus, it is necessary to identify new effective therapeutic strategies for ESCC, especially molecularly targeted therapies, based on a better understanding of the biological events of ESCC cells. Cancer stem cells (CSCs) are a limited number of cancer cells with a self-renewal potential and extensive proliferation capacity and play a dominant role in tumor initiation, metastasis and recurrence (2–4). In pancreatic cancer, a subpopulation of migrating CD133^+^CXCR4^+^ CSCs was reported to be essential for tumor metastasis (5). In glioma, CD133^+^ CSCs were associated with radioresistance and contributed to tumor recurrence after radiotherapy due to preferential activating DNA damage checkpoint response. In malignant melanoma, the drug transporter and chemoresistance mediator ABCB5 was identified as a novel molecular marker for a distinct subset of chemoresistant, stem-cell phenotype-expressing tumor cells, indicating that ABCB5 may be a specific target to enhance cytotoxic efficacy (6). Zhang *et al* also provided evidence that CSC-like cells may play a role in the progression and drug resistance of bladder cancer (7). The aforementioned studies showed that CSCs can be a good therapeutic target for various types of cancer. Although CSCs are common many types of tumors (8–11), the data for CSCs from human ESCC are conflicting.

CD271, a member of the tumor necrosis factor receptor superfamily (12), plays a role in cell proliferation, survival, and apoptosis (13). It is also known as a nerve growth factor receptor and interacts with neurotrophins (14). CD271^+^ cells have been reported to be genuine CSCs in human melanoma (15). CD271^+^ cells showed higher tumorigenecity and metastatic ability in melanoma (16). The high level of CD271 expression was correlated with a poor prognosis for patients with hypopharyngeal cancer (17). Okumura *et al* suggested CD271 as the human esophageal keratinocyte stem cell marker, which may be valuable for prospectively investigating stem cell regulation in association with different biological processes including the neoplastic transformation of regenerative epithelia (18). Authors of that study also identified CD271 as being expressed in 49.2% ESCC and necessary for survival and maintenance of ESCC tumors (19). Huang *et al* demonstrated that CD271^+^ cells possess some characteristics of CSCs (20). However, there is no report with regard to the epigenetic regulation on CD271 expression.

In the present study, we confirmed that CD271^+^ ESCC cells exhibited higher self-renewal ability and chemoresistance. CD271 expression was associated with TNM stage and metastatic capacity in human ESCC and we found that CD271 expression was regulated by DNA methylation. Our results showed that CD271^+^ ESCC cells possess stem-like properties and their expression is epigenetically regulated.

## Materials and methods

### Cell line and tissue specimens

The KYSE70 ESCC cell line was preserved in our laboratory and maintained in RMPI-1640 supplemented with 10% fetal bovine serum (both from Hyclone, Logan, UT, USA), 100 U/ml of penicillin, and 100 μg/ml of streptomycin at 37°C, 5% CO_2_. Sixty-three paired ESCC tissues and adjacent non-cancerous tissues were previously collected and stored (2008–2010). Tissues were provided by the Department of Pathology, The First Affiliated Hospital of Zhengzhou University, with confirmed histopathological results. Information pertaining to clinicopathological parameters were also available.

### Flow cytometric analysis and cell sorting

Adherent KYSE70 cells were trypsinized and dissociated into single cells suspended in PBS with 3% fetal bovine serum. The cells were stained with PE-conjugated mouse anti-human CD271 monoclonal antibody (BD Biosciences, San Jose, CA, USA). The corresponding isotype immunoglobulins were used as controls. Dead cells were identified using 7-AAD (Biolegend, San Diego, CA, USA). Samples were analyzed using BD FACS Canto II cytometer (BD Biosciences) and sorted with a MoFlo XDP cytometer (Beckman, Brea, CA, USA).

### RNA extraction and cDNA synthesis

Total RNA was extracted from KYSE70 cells and tissue specimens by TRIzol reagent (Invitrogen Life Technologies, Carlsbad, CA, USA) according to the manufacturer’s instructions. The first-strand cDNA was synthesized from 1 μg of total RNA using PrimeScript RT reagent kit with gDNA Eraser (Takara, Shiga, Japan).

### Quantitative real-time PCR

The cDNA was used as a template to detect the expression of CD271 in KYSE70 cells and tissue specimens. qPCR was performed using SYBR Premix Ex *Taq* II (Takara) and assessed by Agilent Mx3005P. GAPDH was used as an internal control. The data were analyzed by 2^−ΔΔCt^. Primer sequences for qPCR are shown in Table I.

### Sphere formation assay

The sorted CD271^+^ and CD271^−^ cells were resuspended in DMEM/F12 medium (Invitrogen Life Technologies) supplemented with 4 μg/ml heparin (Sigma, St. Louis, MO, USA), B27 (1:50, Gibco, Life Technologies, Carlsbad, CA, USA), 20 ng/ml EGF, 20 ng/ml basic FGF (both from PeproTech, Rocky Hill, NJ, USA), penicillin 100 IU/ml and streptomycin 100 μg/ml and then seeded in 24-well ultra-low cluster plates (Corning Costar, Corning, NY, USA). After culturing for 7 days, the number of spheres was counted under a microscope (Leica, Wetzlar, Germany).

### Anchorage-independent cell growth

The sorted CD271^+^ and CD271^−^ cells (2×10^3^ cells/well) were suspended in complete medium containing 0.6% low-melting-point agar (Sigma) and then applied to the top of a 1.2% agar/complete medium layer in six-well plates. After 14 days, surviving colonies were stained with 0.4% crystal violet (Sigma) and counted under a microscope (Leica, USA). The experiments were carried out in triplicate wells. Representative images were taken.

### Drug sensitivity assay and chemotherapy resistance assay

5-Fluorouracil (5-FU) and Cisplatin (DDP), both from Sigma, were dissolved according to the manufacturer’s instructions. The sorted CD271^+^ and CD271^−^ cells were seeded at 3×10^3^ cells/well in 96-well plates and treated with chemotherapeutic reagents in quadruplicate. Cell viability was evaluated using CCK-8 assay (Biyuntian, Jiangsu, China) following treatment with chemotherapeutic reagents for 48 h, and the absorbance was measured at 450 nm using Multiskan Mk3 (Thermo Fisher Scientific, San Jose, CA, USA). The percentage of survival in treated cells was normalized with untreated controls.

KYSE70 cells were continuously treated with DDP (0.5 μM) and 5-FU (0.5 μg/ml) until significant DDP and 5-FU resistance in KYSE70 cells was observed via cell viability assessment. Flow cytometry was then used to detect the CD271 expression.

### Bisulfite modification and methylation analysis

Genomic DNA extracted from the KYSE70 cell line using the Takara MiniBEST Universal Genomic DNA Extraction kit (Takara), was modified by sodium bisulfite using the EpiTect Bisulfite kit (Qiagen, Germany) according to the manufacturer’s instructions. Methylation status was analyzed by bisulfite genomic sequencing (BSP) of the CpG islands. The region was amplified using the primers shown in Table I. Amplified products were cloned into pMD-18T simple vector (Takara), transformed into DH5α competent cells (Takara), and plated under ampicilin selection. Five independent clones were sequenced.

### Statistical analysis

Data were expressed as mean ± SD and analyzed using the Student’s t-test. Paired t-test was used for paired samples. Non-parametric test was performed for samples of non-normal distribution. Statistical analyses were conducted with SPSS 17.0 software. P<0.05 was considered to indicate a statistically significant difference.

## Results

### CD271 expression is associated with stage and lymph node metastasis in human ESCC specimens

**Figure 1:**
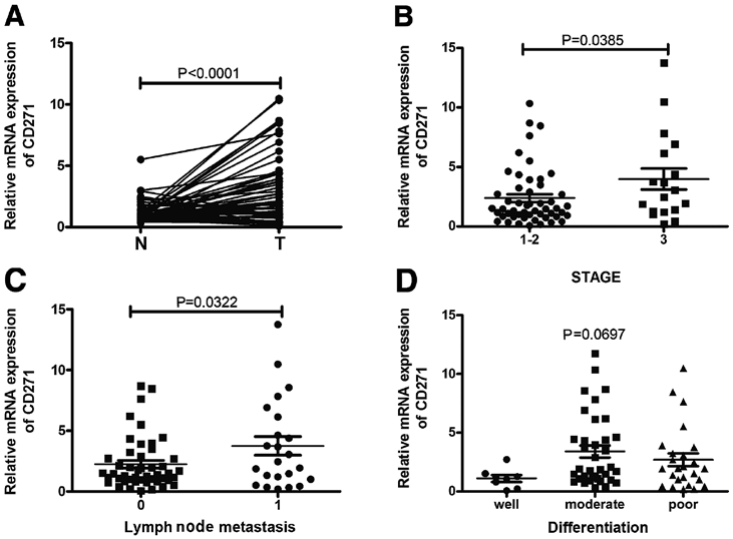
The expression of CD271 is associated with stage and lymph node metastasis in human ESCC specimens. (A) The mRNA expression of CD271 was investigated in paired human ESCC specimens (T) and adjacent non-cancerous tissues (N) by qPCR. The correlation of CD271 expression with clinicopathological characteristics such as (B) stage, (C) lymph node metastasis and (D) differentiation was analyzed.

CD271 is considered crucial to maintain tumorigenecity and stem-like properties of cancer cells including melanoma, hypopharyngeal cancer and esophageal cancer. We compared the expression of CD271 in paired human ESCC specimens and adjacent non-cancerous tissues. As shown in Fig. 1A, qPCR analysis revealed frequent upregulation of CD271 mRNA expression in carcinoma tissues compared with adjacent non-cancerous tissues, indicating that CD271 may act as an oncogene in human ESCC. Moreover, the expression of CD271 was significantly correlated with TNM stage and lymph node metastasis but not other variables such as age, gender and differentiation (Fig. 1B–D).

### CD271^+^ cells overexpress stem-related gene NANOG and EMT markers

**Figure 2:**
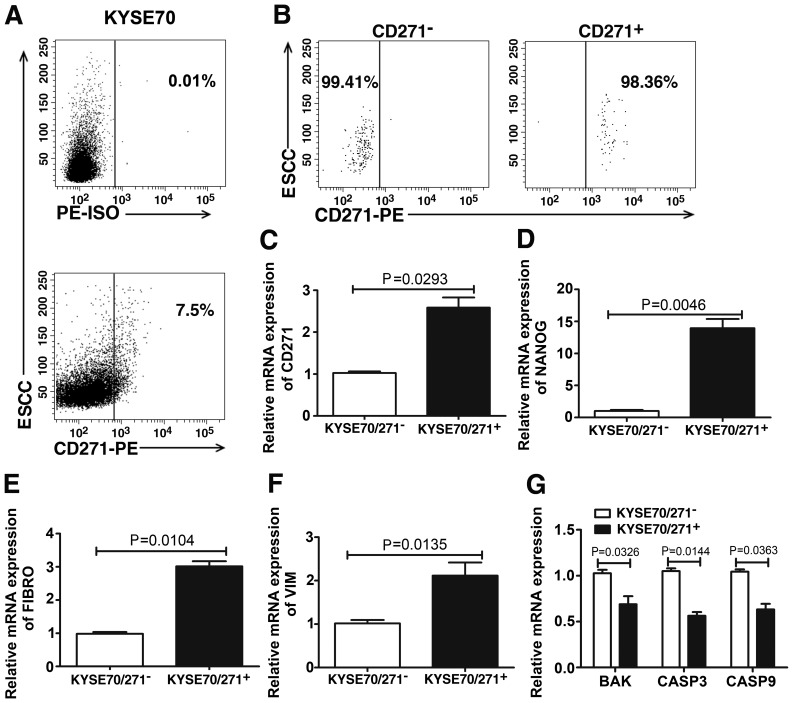
CD271^+^ cells express high levels of stem-related gene NANOG and EMT markers. (A) CD271 expression in KYSE70 was detected by flow cytometry. Of the cells 7.5% intensely expressed CD271. (B) The purity of sorted CD271^+^ and CD271^−^ cells was 99.41 and 98.36%, respectively. (C) Quantitative real-time PCR analysis of CD271 expression in sorted CD271^+^ and CD271^−^ cells. In the two subpopulations, relative expression of (D) NANOG, (E) Fibronectin (FIBRO), (F) Vimentin (VIM) and (G) apoptosis genes BAK, Caspase 3 and Caspase 9 was analyzed by qPCR.

To study the stem cell-like properties of isolated CD271^+^ cells from the KYSE70 ESCC cell line, we first detected the CD271 expression by flow cytometric analysis. We found the CD271^+^ subpopulation was 7.5% present in the KYSE70 cell line (Fig. 2A). CD271^+^ and CD271^−^ cells were then sorted separately and the purity of the two sorted subpopulations was 98.36 and 99.41%, respectively (Fig. 2B). qPCR was used to confirm the expression of CD271 in the two subpopulations (Fig. 2C). We also compared the expression of the stem-related gene NANOG, EMT markers Fibronectin (FIBRO) and Vimentin (VIM), and apoptosis genes BAK, Caspase 3 and Caspase 9 in CD271^+^ and CD271^−^ cells. Our results showed that compared with CD271^−^ cells, CD271^+^ cells exhibited an increased expression of NANOG, FIBRO, VIM (Fig. 2D–F) and a decreased expression of BAK, Caspase 3 and Caspase 9 (Fig. 2G).

### CD271^+^ cells form more self-renewing spheres and promote anchorage-independent growth

**Figure 3:**
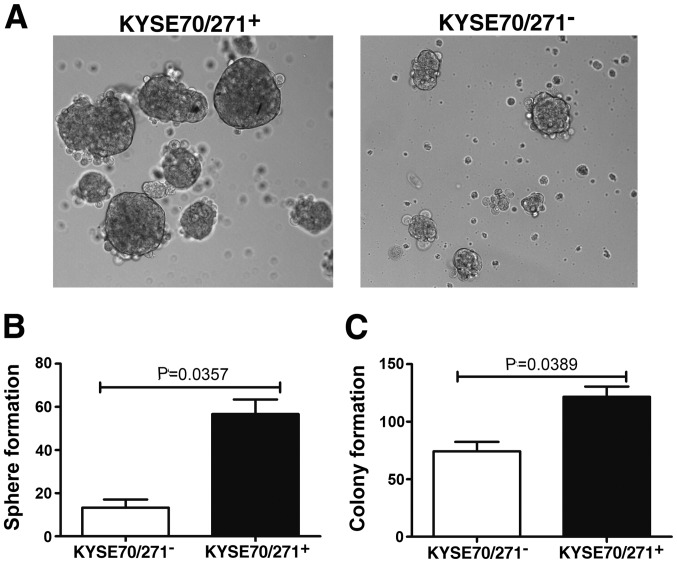
CD271^+^ cells form more self-renewing spheres and promote anchorage-independent growth. (A and B) The capability of tumor sphere formation was measured in sorted CD271^+^ and CD271^−^ cells. (C) Anchorage-independent soft-agar growth was analyzed in sorted CD271^+^ and CD271^−^ cells.

One of the most important properties of GSCs is self-renewal. We examined the tumor sphere formation ability, which represents a self-renewal capacity. The sorted CD271^+^ and CD271^−^ cells were seeded in 24-well ultra-low cluster plates at a density of 5×10^3^ cells/ml in DMEM/F12 medium supplemented with heparin, B27, EGF, and bFGF. After culturing for 7 days, CD271^+^ cells exhibited a marked ability for tumor sphere formation, compared with CD271^−^ cells (Fig. 3A and B). In addition, CD271^+^ cells promoted anchorage-independent growth (Fig. 3C), indicating that the CD271^+^ cells play an important role in the maintenance of malignant growth of ESCC cells. These results suggested that CD271^+^ ESCC cells possess stem-like properties.

### CD271^+^ cells possess the ability to resist conventional chemotherapeutic reagents in vitro

**Figure 4:**
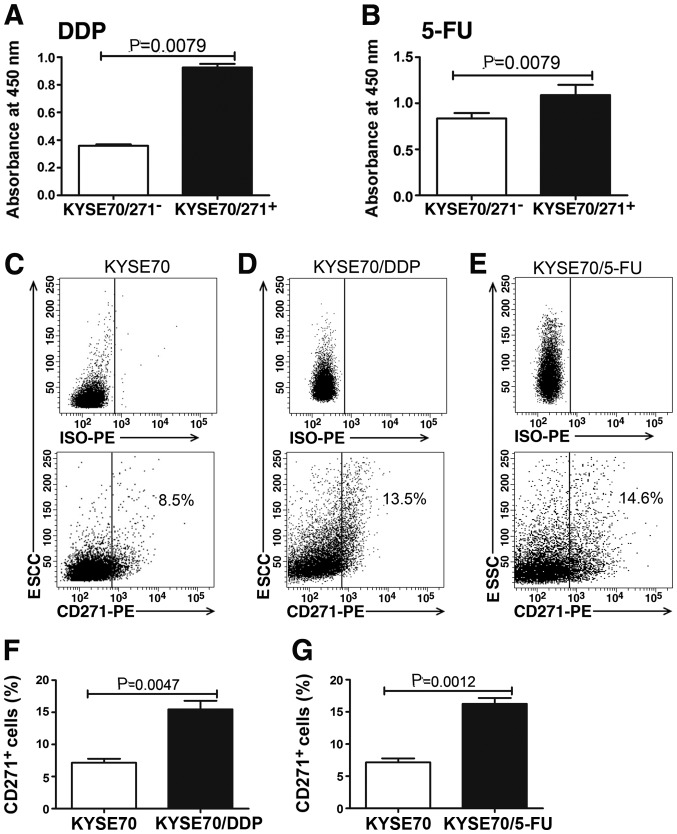
CD271^+^ cells possess the ability to resist to conventional chemotherapeutic reagents *in vitro*. Surviving cells were detected in sorted CD271^+^ and CD271^−^ cells treated with (A) DDP and (B) 5-FU by CCK8 assay. CD271 expression was analyzed in (D and F) DDP-, (E and G) 5-FU-resistant cells and (C) parent cells by flow cytometry.

Cancer stem cells are more resistant to conventional chemotherapeutic drugs. To examine whether the self-renewing CD271^+^ cells possess the hypothesized CSC chemoresistant ability, the sensitivity of the sorted CD271^+^ and CD271^−^ cells to DDP and 5-FU, respectively, was analyzed. The survival rates of CD271^+^ cells were higher under the treatment of DDP and 5-FU, compared with CD271^−^ cells (Fig. 4A and B). We also identified CD271^+^ stem-like cells could be enriched in DDP- and 5-FU-resistant cells (Fig. 4C–G). The results validated a role for CD271^+^ cells in chemoresistance, which may explain the failure of current therapies to eradicate progenitors and prevent tumor recurrence.

### CD271 expression is regulated by DNA methylation

**Figure 5:**
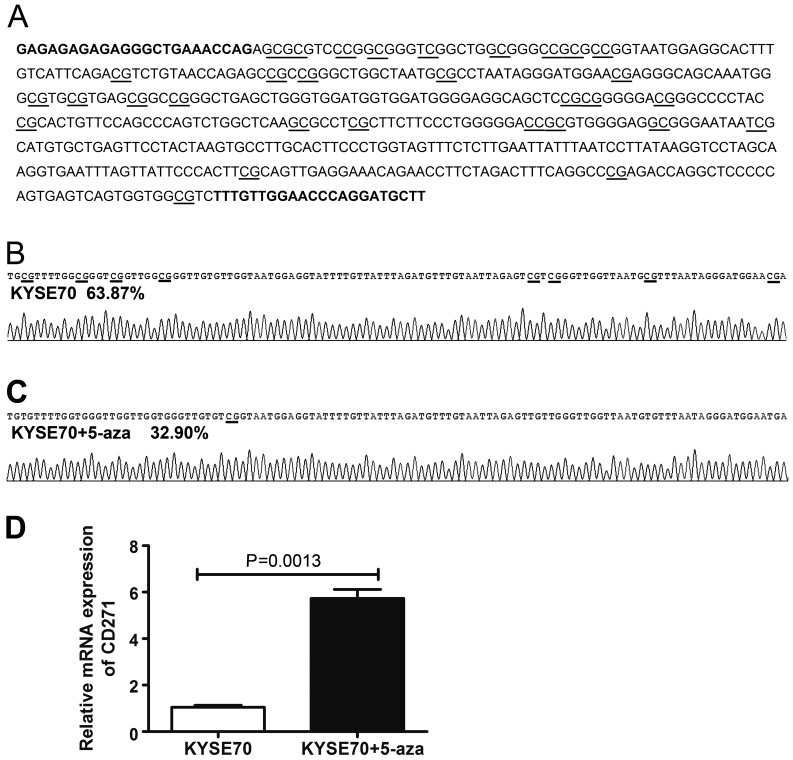
CD271 expression is regulated by DNA methylation. (A) CD271 promoter region. The analyzed CpG dinucleotides are underlined. Primers used for bisufilte genomic sequencing are in bold. Methylation status of 31 CpG sites in the promoter region of KYSE70 cells treated (B) without or (C) with demethylation agent 5-aza. (D) qPCR analysis of CD271 expression in KYSE70 cells treated with or without 5-aza.

Transcription factors and epigenetic modifications often guide external signals to a specific genetic response. We examined whether epigenetics including DNA methylation are involved in regulating CD271 expression. To investigate promoter methylation of CD271, BSP was performed. The area of the CpG-rich region spanning 31 CpG sites, was sequenced (Fig. 5A). We found most CpG dinucleotides were methylated in KYSE70 cells (Fig. 5B). To elucidate whether the methylation of CD271 was associated with its expression, KYSE70 cells were treated with 5-aza (an inhibitor of the methylase enzyme, which can reactivate mRNA expression suppressed by methylation) for 6 days and performed BSP and qPCR to detect the promoter methylation and expression of CD271. Following treatment with 5 μM 5-aza for 6 days, CD271 gene exhibited an obvious induction and the promoter methylation level was reduced (Fig. 5C and D), suggesting that the expression of CD271 is regulated by DNA methylation.

## Discussion

The majority of ESCC patients present with an advanced stage at the time of diagnosis, with poor prognosis, rapid growth and spread due to late diagnosis. Accumulating evidence suggested the CSCs theory in that tumorigenic potential is largely restricted to CSCs (21,22). CSCs from tumor tissues or established cancer cell lines can be isolated by cell-surface markers expressed on CSCs (17,23). In recent studies ALDH was identified as the CSC marker for various types of cancer including ovarian (24), breast (25), lung (26) and prostate (27) cancer, CD44^+^CD24^−^ for breast (28) and ovarian (29) cancer, CD133 for non-small cell lung (30), liver (31) and lung (32) cancer. However, Meng *et al* reported that both CD133^+^ and CD133^−^ subpopulations contain similar numbers of CSCs (33). Therefore, identification of specific cell-surface markers to define CSCs is important for the possible establishment of target-specific therapies using small molecule inhibitors or humanized antibodies.

CD271 knockdown was found to eliminate the capacity of melanoma cells to form heterogeneous tumors most likely through the downregulation of mediators for melanoma invasion and metastasis (GLI-2, SOX2 and ERBB3), angiogenesis (IGFBP-2), proliferation (FST and MITF) or chemoresistance (RHOJ) (34). CD271 was also identified as a predominant molecule responsible for the proliferation, tumorigenecity and plasticity of melanoma cells. Accumulating evidence has shown that metastases develop when distant organs are seeded with CSCs derived from a primary tumor (35). Boiko *et al* identified that CD271^+^ melanoma cells lacked the expression of TYR, MART1 and MAGE genes in 86, 69 and 68% of melanoma patients, respectively, which may explain the reason for T-cell therapies targeting these antigens usually resulting in only temporary tumor shrinkage (16). Okumura *et al* have shown that CD271 expression correlated with negative lymph node metastasis and lower TNM staging. There was no significant correlation between CD271 expression and distant metastasis in ESCC (19). However, in this study we show that the expression of CD271 was significantly higher in ESCC tissues than adjacent non-cancerous tissues. In contrast to findings of a previous study (19), we found that the overexpression of CD271 was significantly associated with TNM stage and lymph node metastasis. Taken together, these data strongly suggest CD271 as an oncogene that plays an important role in ESCC progression.

Cell sorting yielded CD271^+^ and CD271^−^ cells and identified the overexpression of the stem-related gene NANOG in CD271^+^ cells. Since EMT plays a key role in tumor invasion and metastasis during tumor progression, we tested the expression of EMT markers between the two subpopulations. As expected, CD271^+^ cells highly expressed mesenchymal markers, such as FIBRO and VIM. We also found that CD271^+^ cells were able to form more tumor spheres and colonies compared with CD271^−^ cells. Our results further showed that CD271^+^ cells exhibited general resistance to DDP and 5-FU, with higher survival percentages compared with CD271^−^ cells. Taken together, these data suggest that CD271 is a marker of stem-like cells in ESCC. In addition, we observed increased CD271^+^ cells in DDP- and 5-FU-resistant cells. In other words, conventional chemotherapeutic drugs, such as DDP and 5-FU, may selectively decrease the CD271^−^ population, resulting in a relative increase of CD271^+^ cell frequency. Thus, CSCs can be responsible for treatment failures of chemotherapy and poor clinical outcomes.

Accumulating evidence has suggested that CSCs are not only governed by genetic alterations but also aberrant epigenetic regulation. Sun *et al* reported that a unique set of genes such as NANOG, OCT4 and SOX9, were demethylated in invasive cancer cells but methylated in non-invasive cancer cells, indicating that they may be biologically important in the invasive population and upregulated during the EMT process (36). Schirmer *et al* (39) found that the gene L1CAM, which can promote cell motility, invasion and metastasis formation in various human cancers (37–39), could be induced by treatment with the demethylation agent 5-aza in endometrial carcinoma cell lines (40). We previously found that tumor-suppressor gene SPINT2 was induced by a demethylation agent (41). This shows that following epigenetic drug treatment, hypermethylated tumor-suppressor genes as well as oncogenes may be activated simultaneously. In the present study, we firstly found CD271 was regulated epigenetically and its expression could be induced by demethylation agent treatment. Therefore, epigenetic therapy may be a ‘double-edge sword’ and unexpected side effects may occur.

In conclusion, we have demonstrated that CD271, the ESCC cancer stem cell marker, is a potential prognostic marker of patients with ESCC and is regulated epigenetically.

## Figures and Tables

**Table I:** Primer sequences for qPCR for all the genes tested.

Gene name	Sequence	Product size (bp)
qPCR		
GAPDH-F	GCACCGTCAAGGCTGAGAAC	138
GAPDH-R	TGGTGAAGACGCCAGTGGA	
CD271-F	AACAAGACCTCATAGCCAGCA	119
CD271-R	CAGGATGGAGCAATAGACAGG	
NANOG-F	CAAAGGCAAACAACCCACTT	158
NANOG-R	TCTGCTGGAGGCTGAGGTAT	
FIBRO-F	CAGTGGGAGACCTCGAGAAGA	169
FIBRO-R	GTCCCTCGGAACATCAGAAAC	
VIM-F	GAGAACTTTGCCGTTGAAGC	163
VIM-R	GCTTCCTGTAGGTGGCAATC	
BAK-F	CATCAACCGACGCTATGACTC	192
BAK-R	GTCAGGCCATGCTGGTAGAC	
CASP3-F	AGAACTGGACTGTGGCATTGAG	191
CASP3-R	GCTTGTCGGCATACTGTTTCAG	
CASP9-F	CTCAGACCAGAGATTCGCAAAC	116
CASP9-R	GCATTTCCCCTCAAACTCTCAA	
BSP		
CD271-BF	GAGAGAGAGAGGGTTGAAATTAG	505
CD271-BR	AAACATCCTAAATTCCAACAAA	

qPCR, quantitative polymerase chain reaction; BSP, bisulfite genomic sequencing.
